# Design rules for time of flight Positron Emission Tomography (ToF-PET) heterostructure radiation detectors

**DOI:** 10.1016/j.heliyon.2022.e09754

**Published:** 2022-06-18

**Authors:** Philip Krause, Edith Rogers, Muhammad Danang Birowosuto, Qibing Pei, Etiennette Auffray, Andrey N. Vasil'ev, Gregory Bizarri

**Affiliations:** a Cranfield University, Bedfordshire, MK43 0AL, England, United Kingdom; b CINTRA UMI CNRS/NTU/THALES 3288, 637553, Singapore; c University of California, Los Angeles, CA 90095, United States; d CERN, 1211 Geneve 23, Switzerland; e Lomonosov Moscow State University, 119991 Moscow, Russia

**Keywords:** Heterostructured radiation detector, Ultra-fast timing, Positron Emission tomography

## Abstract

Despite the clinical acceptance of ToF-PET, there is still a gap between the technology's performance and the end-user's needs. Core to bridging this gap is the ability to develop radiation detectors combining a short attenuation length and a sub-nanosecond time response. Currently, the detector of choice, Lu_2_SiO_5_:Ce^3+^ single crystal, is not selected for its ability to answer the performance needs, but as a trade-off between requirements and availability. To bypass the current performance limitations, in particular restricted time response, the concept of the heterostructured detector has been proposed. The concept aims at splitting the scintillation mechanisms across two materials, one acting primarily as an absorber and one as an ultra-fast emitter. If the concept has attracted the interest of the medical and material communities, little has been shown in terms of the benefits/limitations of the approach. Based on Monte Carlo simulations, we present a survey of heterostructure performance versus detector design. The data allow for a clear understanding of the design/performance relationship. This, in turn, enables the establishment of design rules toward the development and optimization of heterostructured detectors that could supersede the current detector technology in the medical imaging field but also across multiple sectors (e.g. high-energy physics, security).

## 1 Introduction

Positron Emission Tomography (PET) imaging is now a well-established and key healthcare examination. Its unique capability to provide functional information at the cellular level has become invaluable for early diagnosis and staging of multiple diseases. The majority of current clinical applications are in the management of cancer with increasing and emerging uses in cardiovascular disease, Alzheimer's disease, inflammation and immunology, in addition to having a major role in the development of new drugs and therapies. As such, PET is highly complementary to anatomic imaging such as Computed Tomography (CT) and Magnetic Resonance Imaging (MRI) - nearly all PET scanners are currently combined with a CT scanner in a single gantry. This has and will continue to support a significant increase in the volume of PET scans performed, e.g. due to increased prevalence of cancers, the emergence of new clinical applications (e.g. prostate cancer, theranostics), and new technology such PET/MRI. However, and despite the clear clinical acceptance of PET, there is still a wide gap between the current technology's performance and the clinical end user's needs which could be achieved by establishing ToF-PET imaging as a less invasive, more flexible and high diagnostic power technology.

Core to such effort is the development of enhanced radiation detector materials. Currently materials used as scintillators are not selected for their ability to fully answer the application's needs, but are chosen as a trade-off between requirements, cost and availability. The inability to match needs to detector properties is deeply rooted in the historical approach of using a unique material as the sole energy conversion medium. This inherent conceptual limitation has imposed extremely tight and often conflicting requirements for such materials. While the application needs for ToF-PET detectors are well defined - i) short attenuation length (high capacity for the material, high density and high Z elements, to stop the gamma-rays), ii) ultra-fast response time (sub-nanosecond between gamma-ray absorption and visible photon emission) and iii) decent energy conversion efficiency (light output) -, the material of choice, Lu_2_SiO_5_:Ce^3+^ (LSO) or its variant Lu_1.8_Y_0.2_SiO_5_:Ce^3+^ (LYSO), represents a technical compromise. LSO answers the needs for short attenuation length and light output, but its response time falls short of approaching the sub-nanosecond requirement (~40 ns) (e.g. [1], [2], [3]). The latter is intrinsic to any lanthanide doped material as used in the vast majority of current scintillators. There is no easy workaround, as removing the lanthanide dopant would lead to degradation of the light output while maintaining it would impede the time response.

Two main approaches have been proposed to address the performance limitations of the lanthanide doped single detector material approach: a concept attempting to 1) move away from the use of lanthanide doping and large bandgap materials (e.g. [4], [5]), and 2) develop a family of radiation sensing heterostructures - in which multiple materials work in synergy to achieve the production of ultra-fast photons and short attenuation length (e.g. [6], [7] for example of single crystal functionalization and heterostructure concept and [8], [9], [10], [11] for its application to radiation detector development). This article further presents the concept of heterostructure radiation detector materials and the implications associated with the use of multiple materials in term of inherent scintillation mechanism, and achievable performance.

## 2 Heterostructure concept

### 2.1 Heterostructure components and design

The overall concept of the heterostructure relies on combining at least two different scintillator materials where one compound acts primarily as an absorber, called the matrix in this article, and the other one as an ultra-fast emitter, called the filler in this article. A heterostructure is then uniquely described by defining the matrix materials, the filler material and the geometries of each substructure. The latter can be complex.

**Figure 1:**
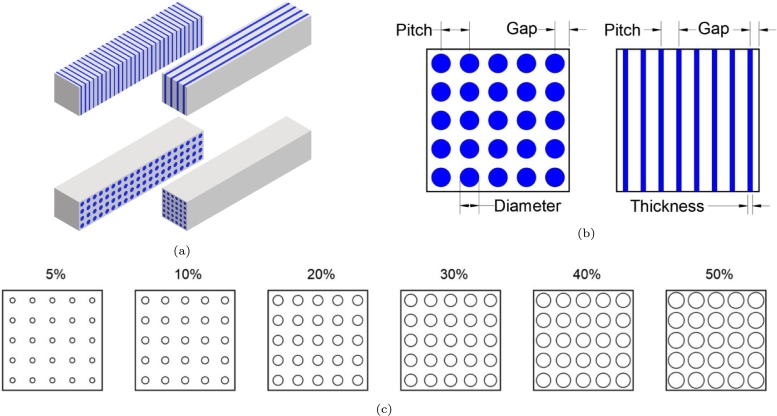
Heterostructure design - a) Plate stacking (upper) and fiber (lower) based structures; b) Parameters associated with each substructure type; c) Filler volume contribution to the heterostructure volume in a fiber based structure.

In this article, we limit the study to rectangular parallelepiped pixels with two main substructure types namely stacking plates (upper drawings in Fig. 1a) and fiber (lower drawings in Fig. 1a) based structures. At this design stage, no requirement for the light collection is introduced allowing designs with substructures aligned along the short or the long axis of the pixel.

The substructure's geometry is defined by three parameters representing the distance between plates/fibers, pitch; the size of the plate/fiber, thickness and diameter, respectively; and the shortest distance between the center of a fiber/plate and an external surface of the pixel, gap (Fig. 1b). To facilitate the comparison and benchmarking of the different heterostructure types, each design is also defined by the volume contribution of the filler component to the overall volume of the heterostructure. Examples of filler volume contribution for a fiber based design is presented in Fig. 1c.

### 2.2 Heterostructure properties

As described by Turtos *et al.* [8], the intended scintillation mechanism in a heterostructure detector material is to maximize the probability of having a photo-electric interaction in the matrix through use of a dense material with high effective atomic number ($Z_{effective}$), followed by the absorption of the recoil electron energy in the filler component which in turn will be converted into prompt photon emission.

Regardless of the choice of matrix/filler materials and heterostructure design, the notion of partitioning the scintillation mechanism with one matrix material largely responsible for the initial absorption and one filler material mostly in charge of the prompt emission of photons leads to fundamental differences in the evaluation of monolithic and heterostructure detectors. In a monolithic single crystal, the light output and timing are uniquely defined by the intrinsic properties of the material and the energy absorbed in the detector. In a heterostructure detector, the partition of the detector of the energy absorbed between the detector substructures implies the notion of fluctuation in the scintillation properties of the detector. From event to event, the energy absorbed in each component of the structure varies leading to a variation of the number of photons created in the matrix and in the filler which in turn results in a distribution of possible light outputs and timing responses. This is a fundamental concept associated with heterostructure detector materials.

### 2.3 Heterostructure performance evaluation

To account for the non-unicity of heterostructure properties, two markers have been defined: 1) the average attenuation length of the heterostructure, called equivalent stopping power in the rest of the article; and 2) a value correlated to the ability of the heterostructure to share the absorbed energy across the two components of the detector, called energy sharing in the rest of the article.

**Figure 2:**
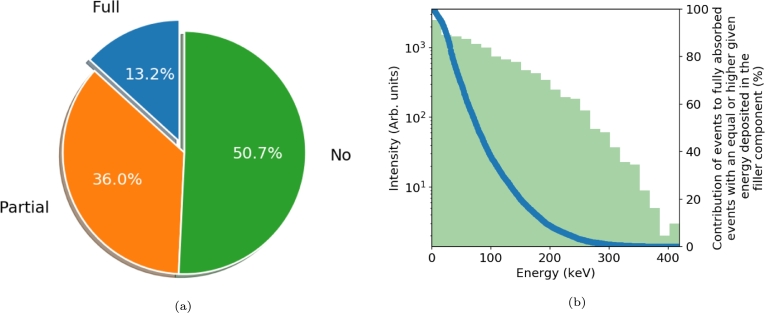
Example of heterostructure performance a) Distribution of fully absorbed events (blue), partially absorbed events (orange) and events that did not interact (green); b) Left axis, Histogram of events resulting from a full absorption of the 511 keV gamma ray as a function of the energy deposited in the filler component (green). Right axis, Contribution to the fully absorbed events with an equal or higher given energy deposited in the filler component as a function of this given energy.

The simulation outputs provide a quantification for both markers. The equivalent stopping power is the number of fully absorbed 511 keV events normalized by the total number of events simulated (Fig. 2a). The energy sharing capability is defined using the spatial information of where the energy has been absorbed. Figs. 2a and 2b show two different representations of the energy sharing capability: 1) an event by event description with the histogram of the fully absorbed events as a function of the energy deposited in the filler component (Fig. 2b - green histogram); 2) an integrated representation of the sum of those events with an energy deposited in the filler component equal or higher to a given energy deposited normalized by the total number of fully absorbed events (Fig. 2b - blue curve). The latter is convenient as uniquely defined when the energy deposited is fixed to a certain value. This allows for easy comparison of the energy sharing capability across different heterostructure designs. In this article, when a single value of the energy sharing is used the threshold was fixed to 25 keV.

This approach allows for both a comparison of individual properties as a function of the heterostructure design but also for the benchmarking of these properties against current ToF-PET pixel technology. The latter is simply done by simulating under the same conditions (pixel dimension, span of 511 keV across the front face) a monolithic single crystal (e.g. LSO or Bi_4_Ge_3_O_12_ (BGO)) and comparing the equivalent stopping power value to the one of the heterostructure of interest. A 3×3×15 mm^3^ LSO and BGO gives an equivalent stopping power of about 36 and 32%, respectively. The energy sharing capability of a monolithic detector is per definition always 100%.

As of now, it is already important to detail what is anticipated for these two properties in the context of heterostructured detectors. For identical geometries, it is expected, in the presence of a low density filler component, that the overall equivalent stopping power of a heterostructured pixel will be lower than the one of a monolithic LSO pixel. There is little to no expectation to increase the equivalent stopping power of a heterostructured scintillator compared to the monolithic equivalent. The overall strategy of the concept is better defined by “Can a lower stopping power be beneficially compensated by the production of fast photons and eventually result in an improvement of the PET performance?”. At this stage, as there is no definite scientific justification of what should be an acceptable decrease of the stopping power, a 20% decrease of the LSO equivalent stopping power is targeted. This would correspond to a value of about 26% for the heterostructure equivalent stopping power.

### 2.4 Heterostructure materials

While modern ToF-PET technology relies to a large extent, if not exclusively, on the use of LSO single crystals, the heterostructure approach opens up options in term of material choice. As for the matrix, if the selection is still mainly dominated by the ability of the material to efficiently stop the incoming radiation, the constraint in terms of fast timing is greatly decreased. Similarly for the filler material, the constraint of having a short attenuation length is partly released and replaced by its ability to generate ultra-fast photons. However the heterostructure approach adds more stringent requirements in term of material structural properties. In order to be assembled according to the heterostructure design, the materials have to be able to be shaped to exact geometries and sustain the potential processing stages associated with it (i.e. precision cutting, polishing and machining operations).

#### 2.4.1 Matrix component

The ideal proprieties for the matrix component are straightforward. The compound, acting as main contributor to the attenuation length of the heterostructure, should be as dense as possible and with a high Z${}_{effective}$. In terms of scintillation properties, the materials only need a decent light output (≃ 10000 ph/MeV) and energy resolution (less than 15%). In terms of machinability, the ideal material should have a low brittleness and a high plasticity.

Amongst commercially available scintillators and despite a fairly open parameter space, only LSO and BGO were found to have a good balance between their scintillation and machinability properties. Both have an excellent attenuation length of 1.15 and 1 cm at 511 keV, respectively. BGO has the highest Z${}_{effective}$ but LSO has a slightly better density. Whilst BGO's structural properties are not ideal, its brittleness index, the ratio between a material's microhardness (H, GPa) and its fracture toughness (K_*C*_, MPa m${}^{\frac{1}{2}}$), is above 7.9 $\mu m^{-\frac{1}{2}}$ ([12], [13]), it does not require any special handling compared to LSO, as lutetium is toxic, and earlier studies by Genov [14] and by Kuzmenko et al. [15] have shown that contact machining of BGO is feasible. The scintillation properties of LSO, as reference material, and BGO are summarized in Table 1.

**Table 1:** Scintillation properties of LSO and BGO scintillators.

Compound	Form	Density (g/cm^3^)	Z${}_{effective}$	Light output (ph/MeV)	Decay time (ns)	Decay time contribution (%)	Rise time (ns)	Ref
LSO:Ce^3+^	Single crystal	7.4	66.4	30000	40	100	0.07	[16]
BGO	Single crystal	7.1	75.2	8200	300	100	0.03	[16]

For completeness, other scintillators have been considered as matrix material candidates but have fallen short of meeting the requirements. The more ductile halides are largely hygroscopic and suffer from a low density and Z${}_{effective}$. Similarly, if oxides such as YAP (YAlO_3_) and YAG (Y_3_Al_5_O_12_) could be considered for their machinability properties, their attenuation length is too limited compared to BGO or LSO. CdWO_4_ at the opposite end of the spectrum has excellent attenuation length but its scintillation and structural properties are crippling. Its brittleness index exceeds 12 $\mu m^{-\frac{1}{2}}$ ([17], [18]), to the authors' knowledge the highest of all the commercial scintillators.

#### 2.4.2 Filler component

The selection criteria for the filler material are heavily skewed toward ultra-fast photon creation ability (ideally lifetime less than 1 ns and rise time in the 10's of picoseconds range). However at this early stage of selection, we have decided not to drastically constrain the scintillation properties requirements and down selected any published compounds with “decent scintillation performance”. Decent performance is here defined by a light output higher than 1000 ph/MeV and a significant contribution of the time response below 10 ns. Table 2 summarizes the properties of six selected materials.

**Table 2:** Scintillation properties of candidate filler compounds.

Compound	Form	Density (g/cm^3^)	Z${}_{effective}$	Light output (ph/MeV)	Decay time (ns)	Decay time contribution (%)	Rise time (ns)	Ref
NE102A / UPS-89	Dye embedded in polymer	1.03	5.87 (PVT)	13000	2.4	100	0.9	i.e. [20]
Cd_*x*_Zn${}_{1-x}$S /ZnS nanocomposite	Core/shell quantum dots and dye (FBtF) embedded in polymer (PVT)	1.865	27.66	9275	6.49	100	0.09	[22]
BaF_2_	Single crystal (Melt growth)	4.89	52.68	11800	0.6; 630	15; 85	0	[16]
(AEIU)PbBr_4_ 2-(2-aminoethyl) isothiourea lead bromide	Single crystal (Solution growth)	3.16	62.4	3000	0.5; 2.2; 6.9	4; 29; 67	0.07^⁎^	[21]
(BA)_2_PbBr_4_ Butyl ammonium lead bromide	Single crystal (Solution growth)	2.44	61.8	40000	1.4; 4.5; 20.8	12; 81; 7	0.07^⁎^	[21]
(PEA)_2_PbBr_4_ Phenethyl ammonium lead bromide	Single crystal (Solution growth)	2.36	59.8	12000	1.1; 5.0; 14.6	2; 22; 76	0.07*	[21]

* This work - Estimated from picosecond pulsed x-ray lifetime measurements.

As for the matrix component, these materials are expected to be able to be shaped to match the intended detector design. For most of these materials with the exception of BaF_2_, their form (polymer), or their synthesis route (solution growth), permit a direct *in situ* synthesis in the matrix sub-assembly. This is a real advantage as it allows for a direct shaping of the filler substructure to requirements. For a single crystal grown from the melt this is not possible and alternative methods have to be used. For BaF_2_, the structural properties of the material are comparable with those of BGO and LSO and allows for manufacturing of thin plates. For fiber based heterostructure designs, the use of the micro-pulling-down technique [19], has been demonstrated to be possible.

## 3 Methods

The simulation of the response of the heterostructure detector was done using the Geant4 framework [23]. Each heterostructure layout was designed in a CAD software (Autodesk Inventor 2019). The overall volume of the pixel was kept constant throughout the study at 3×3×15 mm^3^ varying the geometry of the matrix and/or the filler. The matrix and filler substructures were used as input of the simulation (STL files) and linked to a specific material (i.e. BGO for the matrix).

For each simulation, 50000 511 keV gamma rays were generated along the long axis of the pixel. To account for the spatial non-uniformity of the heterostructure, the incident 511 keV gamma rays were randomly emitted across the front face of the detector (small pixel surface). Each incoming gamma ray and the secondary particles created were tracked throughout the heterostructure to quantify the energy deposited in the matrix and filler components. These data were then used to calculate the equivalent stopping power and the energy sharing capability of the specific heterostructure.

The simulation results were validated against several criteria. The attenuation length was checked against monolithic reference samples (LSO and BGO) and against the standard analytic estimation of the probability of interaction in the crystal. The required number of simulated events to reach a statistically representative result was also checked with a study of the attenuation length and energy sharing capability as a function of the number of initial gamma ray events.

The performance assessment and optimization of the heterostructure design is a multi-variable problem. The parameter space to survey is large with variables such as pitch, diameter/thickness, gap, structure type and filler and matrix materials choice to survey. In addition, the markers associated with good performance are multiple with a search to concomitantly maximize the equivalent stopping power and the energy sharing ability. The survey has been simplified and subdivided into three parts;

• impact of the design on performance by varying the geometry and design type whilst fixing the matrix and filler materials to BGO and plastic scintillator, respectively.
• impact of the filler materials on performance by varying the filler compound and design geometry whilst fixing the matrix material (BGO) and the design type (long axis fiber based design).
• impact of the matrix material on performance by comparing the results obtained from BGO and LSO based heterostructures using two different filler materials (BaF_2_ or (BA)_2_PbBr_4_) and five design geometries while keeping the design type fixed (long axis fiber).

## 4 Results

### 4.1 Impact of the design on individual performance

The influence of the design on the performance was studied by fixing the matrix and filler materials to BGO and plastic scintillator, respectively, and varying the type and geometry of the heterostructure. Two main types of structures were simulated, plate stacking and fiber based designs. Each type was studied for plate stacking or fiber alignment along the long and the short axis of the pixel (1a). The study of the design geometry was done by varying the pitch between fibers or plates from 0.12 to 0.5 mm, the fiber diameter or the plate thickness from 0.006 to 2.5 mm and the distance gap between the pixel external sides and the first plate or fiber, from 0.03 to 2.375 mm. The filler component volume contribution to the total pixel volume, called filler volume contribution, was tracked for each simulated heterostructure.

**Figure 3:**
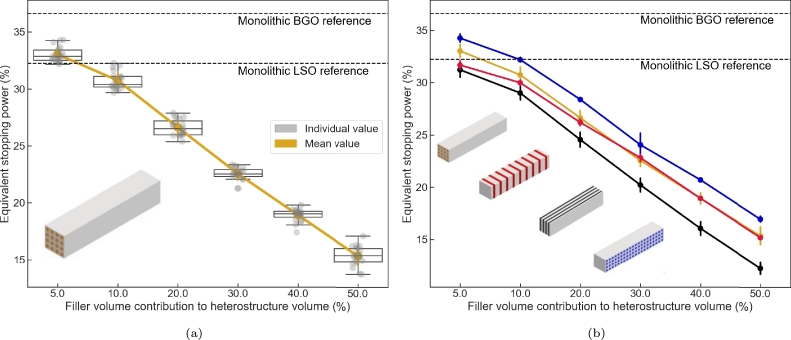
a) Equivalent stopping power for a BGO/plastic long axis fiber based pixel as a function of the filler volume contribution. Each gray circle is a different design geometry. The yellow line links the mean equivalent stopping power averaged across designs for a constant filler volume. b) Mean equivalent stopping power as a function of the filler volume contribution and design type.

Fig. 3a shows the dependence of the equivalent stopping power to the filler volume contribution for a fiber based design aligned along the long pixel axis. Each grey circle represents the equivalent stopping power for a specific pixel design. Also plotted on the graph are the box plots for a fixed filler volume contribution. The yellow line joins the mean value averaged across designs with similar filler volume contribution. Added for reference are the equivalent stopping power values for a monolithic pixel of BGO and LSO (same overall pixel dimensions). The trend is clear. The equivalent stopping power decreases with the increase of the filler volume contribution, the less dense and low Z${}_{effective}$ material. For a fixed filler volume contribution, there is little impact of the design geometry on the equivalent stopping power values. Not presented on the graph is the dependence of the equivalent stopping power as a function of the gap parameter. Within the range studied, the gap between the external side of the pixel and the first substructure, fiber or plate, has little impact on the equivalent stopping power.

The univariable dependence of the equivalent stopping power to the filler volume contribution allows for a direct comparison of the impact of the design type on its value. Fig. 3b presents the mean value of the equivalent stopping power for a fixed filler volume contribution as a function of the four different design types. The spread of the value for a fixed filler volume contribution is presented with vertical error bars. The trends observed previously, dominant role of the filler volume contribution on the achievable equivalent stopping power values and monotonic decrease with increasing filler volume contribution, is seen for the four design types studied. The fiber based structures aligned along the short axis give the best achievable equivalent stopping power values at any filler volume contribution. The worst designs are associated with the stacked plates aligned along the long axis structures. The difference between the best and the worse designs are of about 4% equivalent stopping power value across the entire filler volume contribution range.

**Figure 4:**
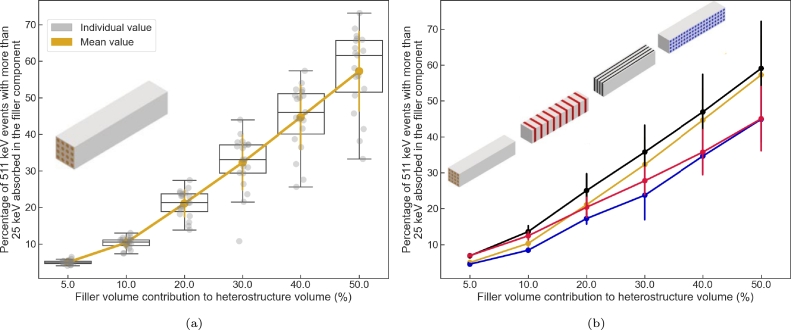
a) Energy sharing (25 keV energy threshold) for a BGO/plastic long axis fiber based heterostructure as a function of the filler volume contribution. Each gray circle corresponds to a design geometry. The yellow line links the mean energy sharing averaged across design with similar filler volume contribution. b) Mean energy sharing values for a BGO/plastic heterostructure as a function of the filler volume contribution and design type.

Figs. 4a and 4b present the impact of design geometry and filler volume contribution on the energy sharing ability of the heterostructure. The variable displayed is the ratio between the number of fully absorbed events with an energy deposited in the filler component of at least 25 keV to the total number of fully absorbed events. Contrary to the equivalent stopping power, the dependence of the energy sharing increases with the increasing filler volume contribution and the design geometry has a large impact on its value as seen in the spread of the data for a fixed filler volume contribution. The latter does not allow the reduction of the dependence of the energy sharing ability to a dominant parameter nor gives a clear picture of the impact of the structure type on the energy sharing ability of the detector (Fig. 4b).

**Figure 5:**
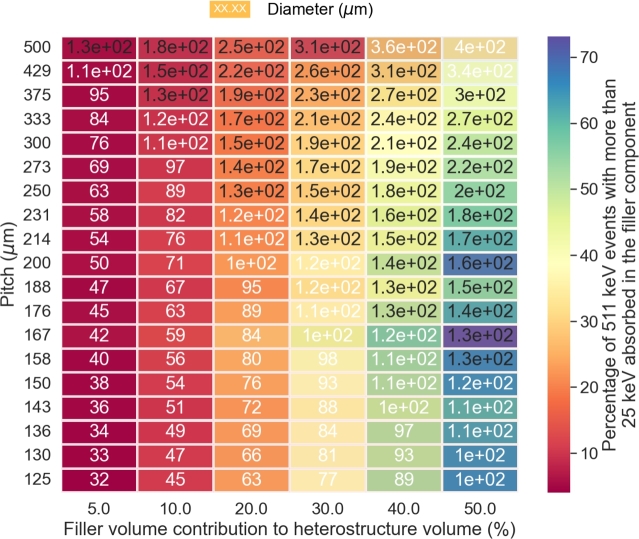
Heat map of the energy sharing capability (25 keV energy threshold) as a function of the filler volume contribution, the pitch and the fiber diameter for a BGO/plastic long axis fiber based pixel.

To further clarify the impact of the heterostructure geometry on the detector energy sharing ability, Fig. 5 presents the same data in a heat map plot with the energy sharing (z axis - colour coded) as a function of the filler volume contribution (x axis), the pitch (y axis) and the diameter of the fiber (numbers displayed in cells). It means that going from one cell to another cell vertically corresponds to a decrease of the fiber diameter. The graph provides a clearer picture of the energy sharing dependence against the geometry of the heterostructure. For a fixed pitch value, the energy sharing increases with the increasing thickness of the filler. For a fixed filler volume contribution, smaller pitch improves the energy sharing between the two components of the heterostructure. These two dependencies are anticorrelated and the optimum values in term of pitch and diameter will vary depending on the stopping power of the filler materials. In the figure and for high filler volume contribution, the optimum pitch/diameter values are found in the middle of the figure.

Similarly to the equivalent stopping power dependence, the variation of the gap parameter within the range studied does not strongly impact the energy sharing ability of the detector.

### 4.2 Impact of the filler material on individual performance

The impact of the filler materials on the heterostructure performance was studied by simulating the response of a long axis fiber based heterostructure fixing the matrix material to BGO and using sequentially as filler material the compounds listed in Table 2. Several geometries have been tested with pitch ranging from 0.1667 to 0.6 mm, fiber diameter from 0.1667 to 0.4873 mm and gap from 0.083 to 0.3 mm.

**Figure 6:**
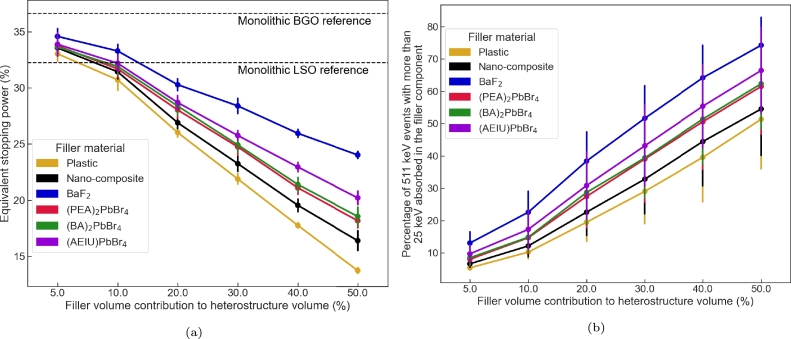
Mean equivalent a) stopping power and b) energy sharing (25 keV energy threshold) values averaged across design geometries of similar filler volume contribution for a long axis fiber based heterostructure (BGO matrix) as a function of the filler volume contribution and the filler material.

Figs. 6a and 6b present the equivalent stopping power and energy sharing capability as a function of the filler volume contribution for the different studied filler materials. For a fixed filler material, each individual trend is similar to the ones observed previously for the BGO/plastic heterostructure. The equivalent stopping power decreases with increasing filler volume contribution. The trend for the energy sharing is anti-correlated with an increase of the energy sharing capability with increasing filler volume contribution. The spread of the data for a fixed filler volume contribution is also comparable to the ones observed previously. In terms of filler material dependence, the best results are obtained with BaF_2_ and the worst with the plastic scintillator.

**Figure 7:**
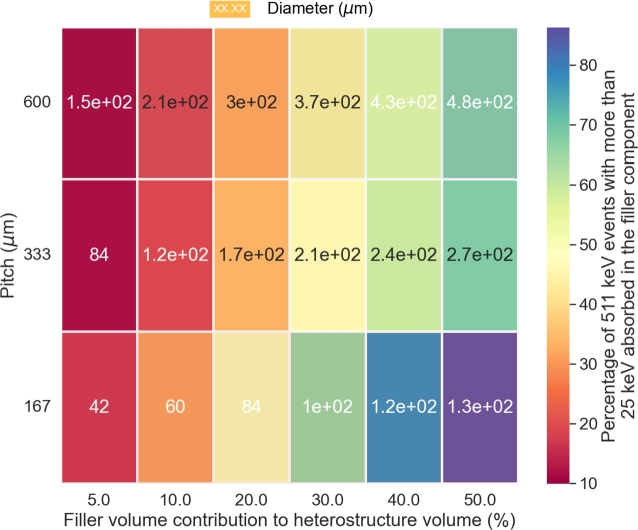
Heat map of the energy sharing capability (25 keV energy threshold) as a function of the filler volume contribution, the pitch and the fiber diameter for a BGO/BaF_2_ long axis fiber based pixel.

For completeness, Fig. 7 shows the heat map of the heterostructure energy sharing capacity (z axis - colour coded) as a function of the filler volume contribution (x axis), the filler pattern pitch (y axis) and fiber diameter (numbers displayed in cells) using BaF_2_ as filler material. The conclusions are similar to the ones observed for the BGO/plastic heterostructure. The energy sharing is maximized for small pitch and large fiber diameter.

### 4.3 Impact of the matrix material on individual performance

The impact of the matrix material on the heterostructure performance was done by comparing the responses obtained from BGO and LSO heterostructures. The simulations were done for a long axis fiber based heterostructure using two different filler materials ((BA)_2_PbBr_4_ and BaF_2_)) and five distinct design geometries.

**Figure 8:**
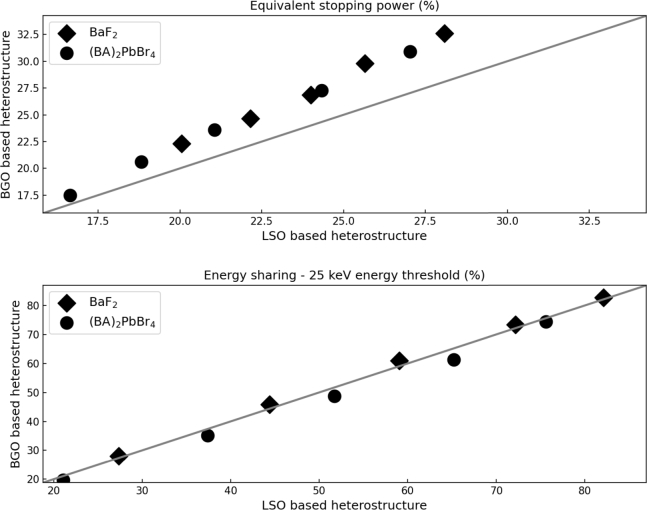
Comparison of the equivalent stopping power (top graph) and the energy sharing (bottom graph) values for a long axis fiber based heterostructure using LSO (x axis) or BGO (y axis) as matrix material for two filler materials (BaF_2_ - diamond and (BA)_2_PbBr_4_ - circle) and five design geometries.

Fig. 8 presents the equivalent stopping power (top graph) and the energy sharing (bottom graph). The x axis gives the values obtained using LSO as matrix material and the y axis the ones simulated using BGO. The grey line represents the linear dependence. The data are represented as a function of the filler materials (BaF_2_ - diamond and (BA)_2_PbBr_4_ - circle) and each marker is a different design geometry.

As expected from the intrinsic properties of BGO and LSO, the results show a better equivalent stopping power ability for the BGO based heterostructures regardless of the filler material or the design geometry used. The difference between the LSO and the BGO structures ranges from about 2 to 4 percent depending on the geometry of the heterostructure. The difference increases with the decreasing filler volume contribution. The energy sharing capability does not change significantly as a function of the matrix materials used for all the configurations studied.

## 5 Modelization and discussion of heterostructured detector properties

The results presented in section 4 cover the impact of a large range of heterostructure parameters against the two main properties, the equivalent stopping power and the energy sharing capability of the pixel detector. The main results are clear: 1) a dominant dependence of the equivalent stopping power on the filler component volume contribution to the total heterostructure volume; 2) a strong impact on the pitch and diameter/thickness on the energy sharing capability of the heterostructure; 3) the superiority of using BGO as matrix material over LSO; and 4) the slight benefit of using short axis alignment structures over long axis alignment design types to increase the equivalent stopping power. These trends were observed across the entire matrix and filler material space studied. More expected was the necessity of using as dense as possible filler materials for good performance. To understand these dependencies and eventually provide guiding rules for development, it is convenient to facilitate the discussion by considering simplified models of the scintillation mechanism in the heterostructure pixel.

### 5.1 Equivalent stopping power

A simple but effective way to understand the impact of heterostructure parameters on the equivalent stopping power is to replace the two components of the heterostructure by an equivalent medium with similar properties. In this approach, the heterostructure can be approximated by an unique material with an attenuation coefficient weighted by the volume fraction of the matrix and filler components. The fraction of gamma ray conversion events can be estimated for both design types (Eq. (1) and Eq. (2) for the short axis and long axis alignment design types, respectively) as a function of the pixel length (L), the volume ratio of the light and heavy components (*ν*) and the attenuation length of the matrix ($\alpha_{heavy}$) and filler ($\alpha_{light}$) materials. Equation (1) corresponds to an equivalent medium approximation and equation (2) to a medium where (1-*ν*) gamma ray photons can propagate in the matrix component only, and *ν* gamma ray photons in the filler component only:

(1) $$f(L)=1-e^{-(\alpha_{heavy}(1-\nu )+\alpha_{light}\nu )L}$$

(2) $$f(L)=1-(1-\nu )e^{-\alpha_{heavy}L}-\nu e^{-\alpha_{light}L}$$

It is important to underline that equations (1) and (2) approximate the fraction of gamma ray converted events and not directly the equivalent stopping power as previously defined. The former is a part of the latter with the probability of the full absorption of a gamma ray being 1) the probability for the initial gamma ray to interact within the detector (fraction of gamma ray converted) and 2) the probability of absorbing the secondary particles created. However, a direct correlation is only possible if the probability of absorbing the secondary particles created is either constant or follows a similar dependence to the fraction of gamma rays converted across the entire parameter space studied.

The dependence of the fraction of gamma rays converted is straightforward. The probability depends on the size of the pixel (mainly length) and on the type of material used (attenuation length). For a specific heterostructure design, these parameters are fixed as is the probability of converting the incoming gamma ray. In terms of secondary particles created and for a 511 keV gamma ray energy, the photo-electric absorption and scattering attenuation coefficients contribute similarly to the total attenuation coefficient of the materials studied; meaning that both scattered gamma rays (scattering event) and recoiled electrons (photo-electric event) need to be considered:

• Following a photo-electric event, the probability of absorbing the high energy electron created depends mainly on the electron density and on the size of the material. The electron density is not directly correlated to the attenuation length and a similar dependence of both functions is unlikely. However, and considering the dimension of the studied pixels (15×3×3 mm^3^), the full absorption of the recoil electron can be approximated to be high and constant. The electron ranges in the materials studied rarely exceed 500 μm for an electron energy of 511 keV (see next section). In this hypothesis, the escape of any fluorescence x-rays generated during the electronic cascade resulting from the photo-electric interaction would be the main parameter that could lead to a non-fully absorbed event. The fluorescence x-rays in the materials studied have typical energies of several 10's of keV which, accounting for the pixel dimensions and the attenuation length at these energies (several order of magnitude higher than at 511 keV), will have a high probability of being absorbed.
• Following a scattering event, the probability of full absorption of the scattered gamma ray is small. This will require at least a photo-electric interaction of the scattered gamma ray which, in view of the pixel dimensions and the likely change in direction of the scattered gamma ray, will have a probability, if not negligible, small enough not to drastically contribute to equivalent stopping power. Any multiple scattering processes leading to full absorption of the 511 keV are even less likely.

In this context, the fully absorbed events are heavily dominated by events resulting from a photo-electric interaction of the incoming gamma rays. As the photo-electric absorption and scattering attenuation coefficients are comparable for a 511 keV gamma ray in the materials presented in Table 1, the equivalent stopping power can be approximated to be equivalent to the fraction of gamma ray converted with a ratio between both values of half. This ratio is, if not exact, consistent with the data simulated for the equivalent stopping power and the ones extrapolated from equations (1) and (2) for the fraction of gamma rays converted.

Another approximation built into equations (1) and (2) is the implication that gamma rays travel parallel to the long axis of the pixel. This is not always correct. Any departure of the gamma ray travel direction compared to the pixel long axis will change the overall media seen by the gamma ray, and in turn its probability of interaction. The latter is more sensitive for the long axis alignment structures and for the fiber based structures.

**Figure 9:**
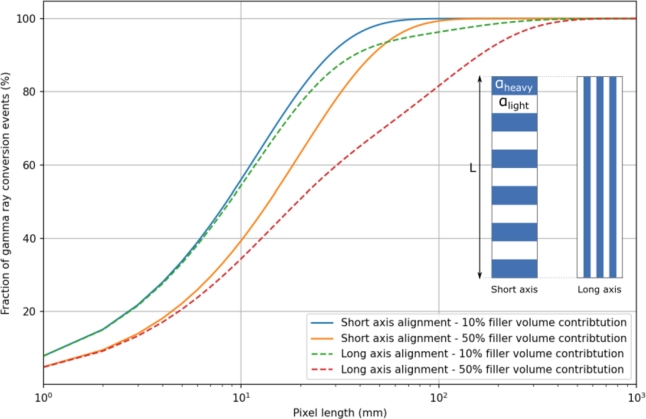
Fraction of gamma ray conversion (equations (1) and (2)) as a function of the pixel length for short and long axis alignment structures. The attenuation coefficients are 1.1 and 10 cm^−1^ for the matrix and the filler components, respectively.

Under these assumptions, Fig. 9 presenting the fraction of gamma ray conversion events calculated from equations (1) and (2) can be used to discuss the impact of the structure parameters on the equivalent stopping power. The data are presented as a function of the pixel length for both structure types and two filler volume contributions to the total heterostructure (10% and 50%). The attenuation coefficient values were fixed at 1 cm^−1^ and 10 cm^−1^ for the heavy and light component materials, respectively. Fig. 9 shows the similarity between the unique material media approximation and the results from the simulation: 1) dominant impact of the filler volume contribution on the equivalent stopping power; 2) impact of the types of heterostructure designs on the equivalent stopping power and the slight benefit of the short axis structures over the long axis ones. The approximation also permits the predicted impact of pixel length on the equivalent stopping power. This is of real importance in terms of the guiding principles of heterostructure pixel development.

**Figure 10:**
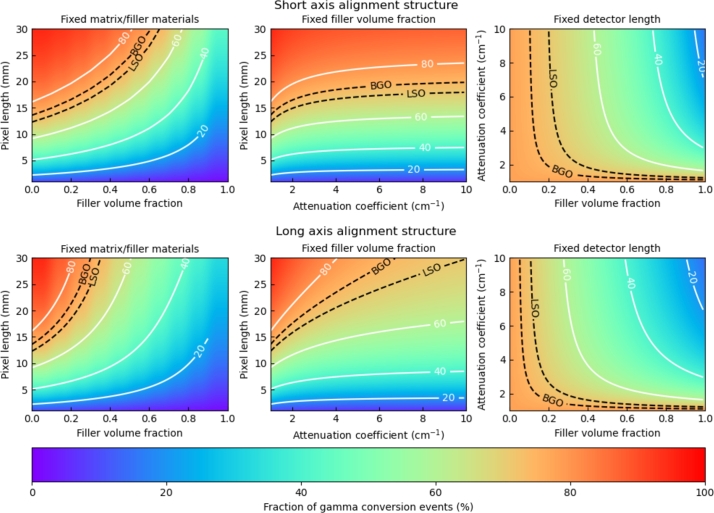
Fraction of gamma ray conversion (equations (1) and (2)) for short (upper graphs) and long (lower graphs) axis alignment structures. From left to right, the graphs represent the fraction of gamma ray conversion as a function of 1) the filler volume contribution and the pixel length with the matrix and filler materials fixed (attenuation coefficient of 1 cm^−1^ for the matrix and of 10 cm^−1^ for the filler material); 2) the attenuation coefficient of the filler materials and the pixel length with the filler volume contribution fixed at 35 %; and 3) the filler volume contribution and the attenuation coefficient of the filler material with the length of the pixel fixed at 15 mm. Added to each graph are two reference isolines corresponding to the fraction of gamma ray converted by a monolithic 3 × 3 × 15 mm^3^ LSO (BGO) pixel.

Comparably to the impact of the pixel length on the equivalent stopping power discussed in Fig. 9, the dependencies of the equivalent stopping power to other parameters can be examined. Fig. 10 presents six nomograms of the fraction of gamma ray event conversion. The three upper graphs are linked to the short axis alignment structures and the lower three to the long axis alignment structures. From left to right, the graphs represent the fraction of gamma ray conversion events as a function of 1) the filler volume contribution and the pixel length with the matrix and filler materials fixed (attenuation coefficient of 1 cm^−1^ for the matrix and of 10 cm^−1^ for the filler material); 2) the attenuation coefficient of the filler materials and the pixel length with the filler volume contribution fixed at 35%; and 3) the filler volume contribution and the attenuation coefficient of the filler material with the length of the pixel fixed at 15 mm. Added to each graph are two reference isolines corresponding to the fraction of gamma ray converted by a monolithic 3×3×15 mm^3^ LSO (BGO) pixel.

These graphs provide additional guidance in terms of heterostructure development and expected performance. Focusing on any specific one will be dependent on the technical choices made and limitations encountered during the manufacturing of the detector. If the choice of the fast component material is the main focus, the middle and right hand graphs will allow for an educated decision based on the expected performance and on the consequences in term of pixel length and/or of filler volume contribution needed to reach the targeted equivalent stopping power value. Similarly, an optimization of the design where the matrix and filler materials are fixed can be guided by the left hand graphs. In addition, the graphs provide a simple way to weight the benefit of a heterostructure design against the equivalent stopping power of a monolithic single crystal pixel.

### 5.2 Energy deposition and energy sharing capability

The discussion of the energy sharing capability of a heterostructure pixel is more complex and cannot be easily approximated by simple equations like for the equivalent stopping power. The energy sharing is heavily dependent, and in a complex way, on the design of the heterostructure as seen in Figs. 4a, 4b, 5, 6b and 7. The energy sharing of the detector is defined by the amount of energy deposited by the recoil electron in both components of the pixel. Understanding and estimating the energy sharing capability of a heterostructure detector is analogous to quantifying this energy deposited as a function of the type of materials used and of the geometry of the two detector components.

**Figure 11:**
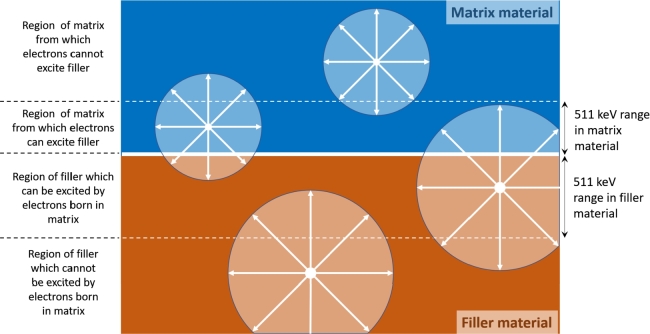
Simplified diagram of a heterostructure detector and the parameters associated with the energy deposition processes.

Fig. 11 presents a simplified 2 dimension diagram of a heterostructure pixel. The design is a stack of matrix (blue) and filler (orange) slices with the matrix material considered as the heaviest one. In term of energy deposition, the volume defined by a sphere of radius equals to the recoil electron range corresponds to the active portion of the heterostructure where the energy is going to be transferred and converted in photons. Depending on the location of the photo-electric interaction, all the energy is going to be absorbed in either one of the components or shared between the two. The energy will only be shared when the distance between the photo-electric interaction occurring in either component (i.e. matrix) to the next component (i.e. filler) is smaller than half of the electron range in the material of the first component (i.e. matrix). In the rest of the discussion, the photo-electric interaction is hypothesised to occur in the matrix component.

**Figure 12:**
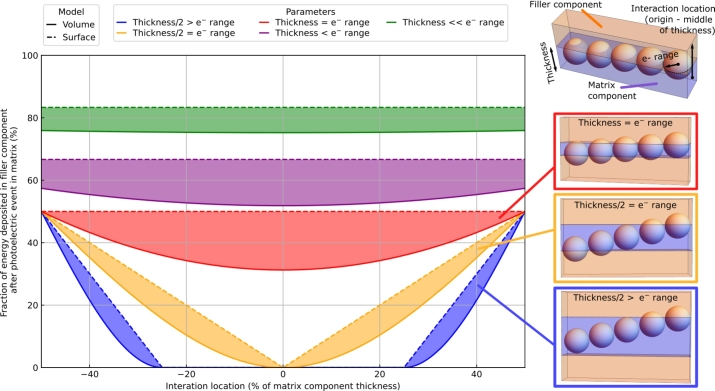
Fraction of energy deposited in the filler component after a photo-electric event in the matrix component as a function of the interaction location and the relationship between the electron range and the thickness of the matrix component.

This simple relationship is effective at linking the geometry of the heterostructure to the energy sharing capability of the detector. Fig. 12 represents the fraction of energy deposited in the filler component after a photo-electric event in the matrix component as a function of the interaction location and the relationship between the electron range and the thickness of the matrix component. Two different models have been used to quantify the fraction of energy deposited in the filler component. The first one, called the volume model, hypothesizes a uniform energy deposited within the volume of the sphere and the second one, called the surface model, uses the approximation that most of energy is deposited at the end of the tracks. For the former, the energy sharing value will correspond to the ratio between the sum of the upper and lower sphere caps volumes and the total volume of the sphere; the sphere caps being defined by the intersection of the electron range sphere and the filler component (see insets in Fig. 12). These two models bracket the achievable energy sharing value of a heterostructure (filled area in Fig. 12). Fig. 12 clearly shows that a good energy sharing capability can only be reached when the thickness of the matrix component is of the order of or smaller than twice the electron range in the matrix material. Focusing only on the energy sharing capability, the smaller the matrix thickness the better, if one accepts the consequences in terms of equivalent stopping power degradation with the increase of the filler volume contribution. Similarly, the model suggests that the thickness/diameter of the filler component should not exceed the electron range in the filler material. Smaller thicknesses will not stop the electron efficiently and larger thicknesses will decrease the overall equivalent stopping power of the detector without additional benefit in term of energy sharing capability. This confirms the simulated data presented previously. Large pitch will prohibit the electron reaching the fibers. For a fixed pitch, the energy sharing is maximized for large fiber diameters as the distance between two fibers decreases and the time spent by the recoil electron within the sub-structure increases. The increase of the energy sharing capability from polymer to BaF_2_ accounting for the large spread of the data due to the design choice could also be explained by an overall increase of the energy deposited within the filler material.

**Figure 13:**
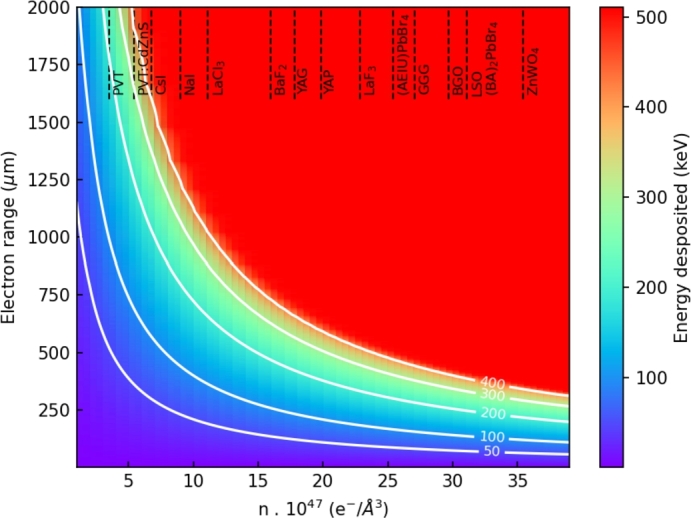
Nomogram of the energy deposited (colour) as a function the type of material (top axis), expressed in terms of electron density (x axis), and electron range (y axis) for a 511 keV electron. Isolines of energy deposited are presented in white.

To further quantify the relationship between electron range and the thickness/diameter of the components, Fig. 13 presents a nomogram of the energy deposited (colour bar) as a function the type of material (top axis), expressed in terms of electron density (x axis), and the electron range (y axis) for an electron of 511 keV energy. Isolines of energy deposited are also presented in white. The energy deposition values were calculated from a modified Bethe formula for low energy electron stopping power [24] accounting for the effective atomic number (Z${}_{effective}$), effective atomic mass (A${}_{effective}$), and density of the material. The latter parameters were also used to quantify the electron density of each material. The electron ranges were estimated from the integration of the energy deposited as a function of the recoil electron energy.

The nomogram adds a layer of quantification to the guidelines introduced previously. Notions previously stated such as “*the thickness of the matrix component is of the order of or smaller than half the electron range*” and “*thickness of the filler component should not exceed the electron range in the filler material*” can now be appraised as a function of the materials used to manufacture the heterostructured detector. As an example a heterostructure with BGO as matrix material should have a matrix component thickness of around 300 μm maximum. The determination of the thickness/diameter of the filler component will depend on the choice of the filler material. It is noteworthy to underline that trying to maximize the energy sharing capability of a BGO/Plastic scintillator design will result in a thickness/diameter of the filler component way higher than 2 mm, an impossibility in view of the standard pixel dimension of 3×3×15 mm^3^ and a resulting equivalent stopping power of less than 10% of a LSO pixel equivalent.

If this relatively simple approach helps to navigate the complex material/heterostructure geometry parameter space and estimates its impact in term of energy sharing capability, it has a certain number of approximations that need to be discussed. The primary ones, are the assumptions linked to the geometry of the electron track. The electron is assumed to uniquely travel forward and in a straight line. If it is a drastic assumption, the discussion presented can be seen as an ideal case of the entire process. The values extracted from Fig. 12 are the upper limits. The non-linear 3 dimensional aspect of the track will only reduce active area where the energy is deposited. Another point that needs attention is the added complexity if the electron encounters multiple cycles of matrix/filler material component along its trajectory. In this case, the sphere approximation presented in Fig. 12 corresponds only to one single step of the process and multiple iterations will be needed in order to extrapolate the design dimensions. Based on Figure 12, Figure 13 this could occur when the matrix thickness and filler thickness/diameter are far smaller than the ranges of the electron in the respective material. For BGO as the matrix material, this will correspond to thickness of less than 50 μm and for a plastic scintillator filler material to a thickness/diameter of about 300 μm.

### 5.3 Further design considerations and developments

In this paper, the emphasis has been on pixel design to maximise scintillation performance through stopping power and energy sharing. These are not, however, the only elements that determine the overall performance of the detector. One area that is strongly influenced by pixel design, geometry and material choice, is light transport. The shape, plate or fiber, and the direction, short or long alignment, of the fast component will have an effect on the light transport and collection, and in turn, on the overall pixel performance. The refractive index mismatch between the different pixel components will lead to partial or total reflectance at boundaries. This will be a major drawback for short axis alignment structures where multiple matrix/filler boundaries will have to be crossed prior to reaching the light detector. A further complication specific to the heterostructure approach is the presence of several emitting media. Compared to the monolithic approach, it will increase the number of light loss pathways through self-absorption within each component (Stoke's shift), but also through absorption of photons from either the light or heavy component by the other (optical cross-talk). This could be mitigated by careful material choice and engineering, i.e. using a wavelength shifting dye for nanocomposite materials, or by optical isolation of the two components, i.e. optical coating.

While the benefit of increasing the pixel length on the equivalent stopping power without reduction of its energy sharing capability was already discussed (equations (1) and (2)), a larger parameter space should eventually be considered in order to fine tune heterostructure performance. One main drawback associated with the simple designs presented here is the strong directional dependence of both energy sharing and stopping power values to the gamma ray and recoil electron trajectories. Consider a plate or fiber of the light component with the heavy component either side. An incident gamma ray or a recoil electron travelling in the direction of the plate or fibre will see only that plate or fibre, whilst one travelling orthogonally to it will interact with both materials. The designs introduced in this study have at most a energy spatial partition along two axes (fiber based designs). More complex designs could offer a degree of compensation and lead to an improved partitioning of the pixel volume. Similarly, other designs could involve less uniform structures with variable pitches and diameters. This could support the fine tailoring of the filler volume. Ultimately, the parameter space for the heterostructured scintillator is vast and consequentially so is the customizability of designs to optimise not only energy sharing and equivalent stopping power but also the mechanisms associated with light transport and collection, machinability, position sensitivity and cost.

Another important criteria to discuss is the potential cost/benefit aspect of the heterostructure approach. Basically, “Will the production cost of these detectors be prohibitive?”. Without entering in a detailed itemization of the production cost, the targeted estimate is to limit the increase of the overall production cost to about 25% for the heterostructure approach compared to the LSO one. This relatively small increase compared to the expected performance gain is mainly justified by the difference in terms of price between the raw material used to develop the presented heterostructures and that of LSO. The detectors part of a standard LSO based ToF-PET can be estimated between 30 and 50% of the total cost of the apparatus. A generic heterostructure is schematically composed of 50% BGO and 50% of a lower cost material (i.e., perovskite compound). This makes the price of the raw material heavily in favour of the heterostructure by a factor of 3 to 4. Of course, the manufacturing cost of the heterostructure will inflate this cost baseline. However, most of the techniques employed to manufacture the heterostructure are already used in large scale production and are not expected to increase the cost by any order of magnitude. Eventually, there is an assumption that these techniques will be further optimized to maximize the production efficiency and minimize the overall detector production cost.

## 6 Conclusions

Whilst the scintillation behaviour of heterostructured detectors is more complex than for monolithic single crystals, by using a simple approach merging simulation and modelling (effective media approximation for the attenuation length to approximate the stopping power and energy deposition for energy sharing) it is possible to establish a series of design rules to guide the development of heterostructured scintillators. To maximise the fraction of fully absorbed events the attenuation lengths of the light and heavy components need to be maximised, the volume ratio between light and heavy elements needs to be minimised, whilst to maximise the energy deposited within the fast component, the pitch between plates and fibres needs to be reduced whilst the thickness/diameter of the fast component is maximised. Whilst the minimisation of the volume fraction and the maximisation of the fast component diameter are in opposition, they can be mitigated through choice of light and heavy components with higher material and electron densities such as BGO for the matrix and BaF_2_ or (BA)_2_PbBr_4_ for the filler, and also through maximising the pixel length. If these simple relationships are effective at understanding the link between the geometry of the detector performance, the modelling of these relationships with simple analytical formula and graphical nomograms enables a real physics-based approach to the development and performance optimization of the heterostructured detectors. While the guidelines are accurate enough to provide quantification, they are also flexible enough to adapt to the problem facing the reader; fine tuning of the geometry when the matrix and filler materials have already been determined (Figs. 10 upper and lower left and Fig. 13) or material candidate screening for a fixed design (Figs. 10 upper and lower middle and right; Fig. 13).

If these results are a real step forward in the possibility to guide the development and engineering of heterostructure detector performance, it is also important to mention that ToF-PET will only benefit from a joint maximization of both the equivalent stopping power and the energy sharing capability of the detector. This is not straightforward as their dependencies on the design parameters are for the most part anti-correlated. This could be solved by establishing an overall Figure of Merit for ToF-PET heterostructured detector performance linking both the equivalent stopping power and energy sharing capability.

Overall the design guidances proposed here provide a physics based approach to the development of heterostructure detectors. This could have some direct and substantial impact in the field of medical imaging but could also be extrapolated to other sectors of activity. There is a considerable interest and economic opportunities for advanced detection materials to support multiple societal grand challenges (e.g. energy with civil nuclear, security with nuclear threats, next generation of detector for energy physics).

## Declarations

### Author contribution statement

Philip Krause: Performed the experiments; Analyzed and interpreted the data; Wrote the paper.

Edith Rogers: Analyzed and interpreted the data; Wrote the paper.

Muhammad Danang Birowosuto, Qibing Pei, Etiennette Auffray: Contributed reagents, materials, analysis tools or data.

Andrey N. Vasil'ev: Analyzed and interpreted the data.

Gregory Bizarri: Conceived and designed the experiments; Performed the experiments; Analyzed and interpreted the data; Contributed reagents, materials, analysis tools or data; Wrote the paper.

### Funding statement

This work was supported by the UK 10.13039/501100000266Engineering and Physical Sciences Research Council (EPSRC) (grant EP/S013652/1 for Cranfield University), the 10.13039/100012470CERN Budget for Knowledge Transfer to Medical Applications and by the 10.13039/501100006769Russian Science Foundation (grant 21-12-00219).

### Data availability statement

Data underlying this study can be accessed through the Cranfield University repository (CORD) at: https://doi.org/10.17862/cranfield.rd.2013173. Data are available under the terms of the Creative Commons Attribution 4.0 International (CC BY 4.0).

### Declaration of interests statement

The authors declare no conflict of interest.

### Additional information

No additional information is available for this paper.
